# A survey of the burden of allergic rhinitis in Hungary from a specialist’s perspective

**DOI:** 10.1186/2049-6958-7-49

**Published:** 2012-11-30

**Authors:** Mária Szilasi, Gabriella Gálffy, Károly Fónay, Zsuzsa Márk, Zoltán Rónai, Zsuzsanna Szalai, Magdolna E Szilasi, Marianna Budai, Veronika Müller, Attila Somfay, Ildikó Horváth, Lilla Tamási

**Affiliations:** 1 Department of Pulmonology, University of Debrecen Medical and Health Science Center, Nagyerdeikrt. 98, Debrecen, H-4032, Hungary; 2 Department of Pulmonology, Semmelweis University, Diósárok 1/c, H-1125, Budapest, Hungary; 3 Elisabeth Teaching Hospital, Győriút 15, H-9400, Sopron, Hungary; 4 Institute for Respiratory Medicine, Munkácsy Mihály u.70, H-2045, Törökbálint, Hungary; 5 Golden Chalice Health Center, Citrom u. 10, H-7621, Pécs, Hungary; 6 KarolinaHospitalMosonmagyaróvár, Pulmonology and Allergology Department, RégiVámháztér 2-4, H-9200, Mosonmagyaróvár, Hungary; 7 Department of Pulmonology, University Clinic Salzburg, Müllner Hauptstraße 48, A-5020, Salzburg, Austria; 8 Department of Pharmaceutics, Semmelweis University, Hőgyes E. u. 7, H-1092, Budapest, Hungary; 9 Department of Pulmonology, University of Szeged, Albert Szent-Györgyi Clinical Center, Alkotmány u. 36, H-6772, Deszk, Hungary

**Keywords:** Allergic rhinitis, Cross-sectional survey, Impact on quality of life, Symptom severity

## Abstract

**Background:**

The casual and severity distribution of allergic rhinitis (AR) in Hungary is unknown.

The aim of this survey was to evaluate symptom perception, disease severity, concomitant asthma frequency and the impact of AR on everyday life activities in a cross-sectional, multicenter study in Hungary under the supervision of Hungarian Respiratory Society.

**Methods:**

Data were recorded by 933 AR patients (65.93% women) and their treating specialists. The perceptions of patients regarding the symptoms (nasal, ocular and others) of AR and its severity, together with its impact on everyday life were assessed. Physicians recorded data regarding the diagnosis and severity of AR, and comorbidities.

**Results:**

52.5% of patients suffered from seasonal AR, 35.1% from perennial AR. A large proportion of patients had moderate to severe disease (MS-AR) (57.34%), persistent disease (98.0%) and concomitant asthma (53.32% in the mild, 57.52% in the MS-AR group). MS-AR was more frequent among women. Despite the treatment used, in MS-AR the proportions of patients reporting moderate to severe rhinorrhoea, nasal obstruction, ocular itching/redness, watering, itchy throat and sneezing were as high as 52.0%, 54.0%, 33.8%, 26.5%, 44.0% and 31.2%, respectively. Overall, there was a poor agreement between disease severity reported by patients and specialists. The adherence to oral antihistamines and intranasal corticosteroids was found to be between 50 and 65%; mostly depending on the dosage form.

**Conclusions:**

AR remains a significant health problem in Hungary because of the burden of symptoms, high rate of concomitant asthma and the significant proportion of MS-AR affecting general well being.

## Background

The incidence and prevalence of allergic rhinitis (AR) has increased worldwide over the past decades. Presently, AR is the most common allergic respiratory disorder, affecting about 10-30% of the general population worldwide, depending on the area and on the age of patients. About 19% of the general population in Europe, while 8.8% to 16% in the United States of America suffers from mild or moderate-severe AR [1,2]. The disease is associated with limited or severe incapacitating symptoms that can affect health-related quality of life, leisure activities, and work productivity even though it responds effectively to treatment in most cases [3-5]. AR is frequently associated with comorbidities such as asthma, sinusitis, otitis media or bridge warp and the coexistence of asthma and allergic rhinitis is characterized by a more severe clinical presentation [6,7]. Between 20% and 50% of patients with AR have asthma, and 30% to 90% of patients with asthma have concomitant AR [8,9]. The severity of AR may be mild or moderate to severe based on its interference with normal sleeping, daily activities, work and school performance [10].

Although the prevalence of AR is increasing in Hungary according to some national epidemiological data [11], there are no data either on its casual and severity distribution or on the rate of its association with asthma. The aim of the present study was to identify the perception of symptoms and the impact of AR on everyday life activities, together with the evaluation of disease severity, concomitant asthma frequency and causative allergens in a cross-sectional, multicenter survey in Hungary.

## Methods

The present multicenter cross-sectional study was initiated and supervised by the Hungarian Respiratory Society and was conducted with Hungarian National Ethical Board approval. All the counties in Hungary were represented with sample sizes proportional to respective general population sizes. The perceptions of patients and physicians regarding the symptoms of AR and its severity and impact on general well being and everyday life were assessed. All the attending doctors were specialists, including allergy specialists, ear, nose and throat doctors and respiratory physicians, and they enrolled the AR patients consecutively on the occasion of patients’ regular visits. AR was diagnosed by the physicians, and adult patients were targeted. Physicians recorded data relating to patient characteristics, diagnosis, AR severity, common triggers, comorbidities, current and past drug treatments and smoking history.

Data were recorded for 933 patients suffering from AR between June 1^st^, 2009 and October 1^st^, 2009. It must be noted that a patient number above 1,000 was targeted; however 74 surveys were either not completely filled out or were not filled out at all, so these surveys were not used in the data analysis. 65.93% women (N=615) and 34.07% men (N=318) were enrolled (Table 1). Mean, standard deviation and median age of patients were 41.93, 14.67 and 41, respectively; ages were between 12 and 79. The 25^th^ and 75^th^ percentiles of the age distribution were 30 and 54 years (as some physicians participating in the study attended also children, few patients below the age of 18 years were also enrolled to maintain the consecutiveness of the enrollment). Diagnostic skin prick tests to confirm AR had been performed on 97.18% of patients at least since the diagnosis. Allergen specific IgE antibody tests were carried out in 22.86% of patients. 14.6% of the patients were smokers, 19.42% ex-smokers, while 65.93% non-smokers. Patients’ characteristics are summarized in Table 1. Physicians completed a patient record form for each patient, and patients were invited to complete a self-completed form.

**Table 1:** General characteristics of the enrolled patients with allergic rhinitis (AR) (persistent disease defined as symptoms experienced on > 4 days/week and for > 4 consecutive weeks)

**Total number of patients**	**933**
Sex, % (n)	Male: 34.07% (318)
	Female: 65.93% (615)
Age, yrs (mean ± SD)	41.93 ± 14.67
Seasonal/perennial/mixed or unknown etiology (%)	52.5 / 35.1 /12.4
Mild AR (%)	42.66
Moderate to severe AR (%)	57.34
Persistent AR (%)	98.0

Data regarding the presence and severity of AR symptoms as well as the impact of AR on everyday life activities were reported by the patients using a questionnaire which was developed by the authors based strictly on Allergic Rhinitis and its Impact on Asthma (ARIA) guideline [10] for this study. Patients recorded information on disease history, symptoms and their severity, the impact of AR on normal activities and treatment adherence. Furthermore, potential factors having an impact on the level of AR control were evaluated by a supplementary questionnaire developed by our research group. Among the typical AR associated symptoms nasal, ocular and others were taken into consideration. As nasal abnormalities: rhinorrhoea and nasal obstruction; as ocular complaints: ocular itching/redness and watering; as other symptoms: sneezing and itchy throat were rated using a score system of 0–3 for each symptom, where 0 *=* no symptoms*,* 1 *=* mild symptoms*,* 2 *=* moderate symptoms and 3 *=* severe symptoms. Thus, the total of nasal, ocular and other scores could have been between 0 and 6, where the higher was the total score the most severe was the symptom. Besides the physicians’ evaluation of the severity of AR (whether it was mild or moderate to severe according to ARIA guideline) [10], the disease severity was also recorded based on patients’ opinion. Patients’ records on the presence of abnormal sleep, impairment of daily activities (sport, leisure), impaired work and school productivity or troublesome symptoms caused by AR were also analyzed (as AR severity determinants according to ARIA guideline). Adherence to medications prescribed for AR was recorded by patients. Missed doses of various treatment forms in the last month were rated as three times or more, once/twice, or never. The optimal adherence was considered if no doses were missed in the last month before the study. The informed consent was obtained from all participants.

### Statistics

In the present study Student’s-t test was used and a p value of < 0.05 was considered significant. To assess any association between AR severity and sex we used the chi-square (χ^2^) method.

## Results

### Patients’ characteristics

According to the physicians’ assessment, 42.66% of patients had mild and 57.34% moderate or severe disease. 52.5% of patients suffered from seasonal, while 35.1% from perennial AR, and in 4.1% of patients both types of AR were identified (for 8.4% of patients there were no data). Aeroallergens most frequently involved in seasonal atopic sensitization were ragweed, mugwort, and birch, while the most common cause of perennial disease was house dust mite. Overall, 98.0% of the patients surveyed had persistent disease (defined as symptoms experienced on > 4 days/week and for > 4 consecutive weeks).

Physicians reported on the cause of visits. Most patients (49.5%) were consulting for repeat prescriptions, 30.9% for routine follow up, and 14.1% because of worsening symptoms (5.5% of patients did not answer the question regarding the cause of doctor visit). Comparing the groups of mild AR and moderate-severe AR, the causes of physician visits were markedly different. In case of mild AR only 1% of patients were consulting due to symptom worsening and 34% for routine follow up. On the contrary, regarding moderate-severe AR patients 22.4% visited their specialist because of disease deterioration and only 22% for routine follow up.

Severity of AR was gender related. While among mild AR patients only 61.23% were women, in case of moderate-severe AR 69.23% of the patients were woman, pointing on higher prevalence of moderate-severe AR among women (χ^2^< 0.05). The mean ages were not significantly different between the two severity groups (41.17±14.87 vs. 42.46±14.52 years, mild vs. moderate/severe, respectively; p > 0.05). The frequency of food cross-allergy was found to be higher among moderate to severe AR patients (23%) than in the subcategory of mild AR (16%). AR had been diagnosed 5.2 years before in mild AR, and 7.0 years before for moderate-severe patients at the visit.

Comparing the assessment of disease severity of physicians and patients - using two-sided t-test with a significance level of 0.05 - it was found that patients rated their disease differently from the physicians. Among patients having mild disease according to the physicians’ assessment, 16.28% rated their disease as moderate/severe. On the other hand, among patients estimated moderate/severe by physicians, 38.04% rated their disease as mild.

### Symptomatology

Table 2 shows the total scores for nasal (rhinorrhoea and nasal obstruction), ocular (ocular itching/redness, watering) and other (sneezing and itchy throat) symptoms and symptom perception according to disease severity (based on physicians records). In the moderate to severe AR group total scores regarding all symptoms were rated higher than in mild AR patients (all p < 0.05). Despite the treatment used, in moderate to severe AR group the proportion of patients reporting moderate to severe rhinorrhoea and nasal obstruction was as high as 52.0% and 54.0%, respectively (Table 3). Furthermore the proportion of subjects with marked ocular itching/redness, watering, itchy throat and sneezing was 33.8%, 26.5%, 44% and 31.2%, respectively in this group.

**Table 2:** Severity of nasal (rhinorrhoea and nasal obstruction), ocular (ocular itching/redness, tearing) and other (sneezing and itchy throat) symptoms were rated using a 0–3 categorical scale, where 0 = no symptoms, 1 = mild symptoms, 2 = moderate symptoms and 3 = severe symptoms

	**Mild AR**	**Moderate-severe AR**
	**Total score (mean ± SD)**	
Total nasal	0.88 ± 0.83	3.02 ± 1.51
Total ocular	0.49 ± 0.77	2.06 ± 1.64
Total AR	1.68 ± 1.48	6.11 ± 2.95

In case of mild AR and moderate-severe AR patients the total nasal and total oral scores (between 0 and 6) for nasal and ocular symptoms are given as mean ± SD. Furthermore, the sum of total scores for nasal, ocular and other symptoms is given as total AR symptom score (between 0 and 18) (mean ± SD). All p < 0.05.

**Table 3:** Frequency (%) of various symptoms in the mild and moderate/severe AR groups

**Type and severity of symptoms**			**Severity of AR**	
			**Mild AR (%)**	**Moderate/Severe AR (%)**
Nasal symptoms	Nasal obstruction	No	55.5	14.6
		Mild	44.5	31.3
		Moderate	0.0	41.8
		Severe	0.0	12.2
	Rhinorrhoea	No	56.1	12.2
		Mild	43.9	35.8
		Moderate	0.0	41.0
		Severe	0.0	11.0
Ocular symptoms	Ocular itching/redness	No	71.3	26.1
		Mild	28.7	40.0
		Moderate	0.0	27.4
		Severe	0.0	6.4
	Watering	No	80.2	38.3
		Mild	19.5	35.2
		Moderate	0.0	21.5
		Severe	0.3	5.0
Other symptoms	Sneezing	No	57.6	13.3
		Mild	42.4	42.7
		Moderate	0.0	33.8
		Severe	0.0	10.2
	Itchy throat	No	68.8	30.7
		Mild	30.9	38.1
		Moderate	0.0	26.1
		Severe	0.3	5.1

### Impact on sleep and daily activities

**Figure 1:**
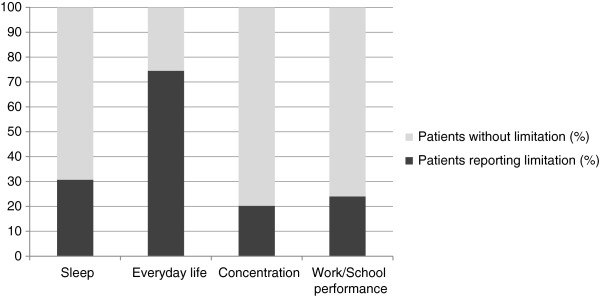
Proportion of patients reporting sleep disturbances, everyday life limitations, concentration problems, altered work or school performance due to moderate to severe allergic rhinitis in Hungary.

In mild AR the disease impact on normal everyday activities and sleep was negligible. On the contrary, for approximately 30.7% of moderate to severe AR patients, the symptoms of AR had a significant impact on sleep patterns or caused sleep disturbances, despite the prescribed AR medications. The majority of the moderate to severe AR patients reported that their symptoms had an impact on daily activities, 24.0% of them reported reduced work/school performance, while 20.2% noticed poor concentration ability. Summarizing the impact of AR on daily activities, 66.0% of moderate to severe AR patients reported mild difficulties, while 8.5% of them had remarkable difficulties because of AR (Figure 1).

### Comorbidities

**Figure 2:**
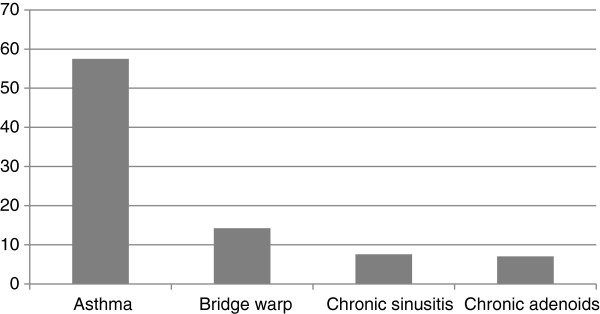
Proportion of moderate to severe allergic rhinitis patients with various concomitant diseases in Hungary.

AR itself is not a life-threatening condition, however, it disposes to some comorbidities, such as sinusitis, otitis media, bridge warp etc., and it is a strong risk factor for the development of asthma. The prevalence of asthma was found to be 53.32% in the mild, while 57.52% in the moderate to severe AR group. Based on otolaryngologic examination which was performed in 67.63% of enrolled moderate to severe AR patients, the prevalence of other comorbidities was determined, too; (data are shown in Figure 2).

### Treatment adherence

The patients used oral antihistamines and intranasal corticosteroids according to the physicians’ prescriptions. All the mild AR patients were prescribed oral antihistamines (cetirizine, levocetirizine, fexofenadine, loratadine or desloratadine) and patients with moderate to severe disease were on both oral antihistamine and intranasal steroid (momethasone, budesonide, fluticasone-propionate or -furoate) therapy in most cases. The optimal adherence regarding antihistamine tablets was reported to be as low as 64.32% for mild and 60.19% for moderate-severe AR patients, while regarding intranasal corticosteroids 50.15% and 50.22% for mild and severe AR group, respectively. Evaluating the possible causes of non-adherence the following were identified: 39.68% of mild AR and 31.49% of moderate-severe AR patients were without symptoms, thus they did not take the tablets. The respective values for nasal corticosteroids were 88.66% and 64.29%. The second most frequent reason of non-adherence with oral treatment was forgetting to take it: this was recorded in 40.48% and 35.91% of mild and moderate/severe AR patients, respectively. Of note, regarding intranasal steroids, this reason was rarely indicated. Side effects were the causes of non-adherence only in 2-4%.

## Discussion

This cross-sectional, multicenter survey was the first to evaluate the severity, impact on everyday life activities, symptom perception, and concomitant asthma frequency in allergic rhinitis patients in all counties of Hungary. Conducted among 933 patients with AR presenting to their specialist, the study found that AR was mostly confirmed with diagnostic tests in Hungary. A marked proportion of patients had moderate or severe disease (57.34%), persistent symptoms (98.0%) and comorbidities such as asthma (53.32% in the mild, while 57.52% in the moderate to severe AR). Underrepresentation of patients with intermittent disease might be the consequence of over-the-counter freely available antihistamine tablets available for intermittent AR patients for 4 weeks without medical prescription.

As reflected in this and other surveys, AR imposes a substantial burden on patients regarding everyday life limitations and work performance [12,13]. Nearly 60% of AR patients had moderate to severe disease, and these patients – although treated – showed marked nasal and ocular allergic rhinitis symptoms and hence suffered from sleep- and concentration disturbances, limitations on everyday life activities, and poor work/school productivity. Majority of moderate to severe patients in this survey considered that they had remarkable difficulties because of AR. However, patients with mild AR did not show limitations regarding any aspects of everyday life.

Our results confirm higher rate of seasonal AR and are similar to those of AILA study (Allergies in Latin America), revealing that 62% of Latin American AR patients are seasonally affected, with the remaining 38% suffering year-long symptoms [14]. In the study of Navarro et al. [9] 46% of cases were intermittent, 54% persistent, 43% mild, and 57% moderate-severe. Pereira et al. [15], whose study included 3225 subjects attending the allergy clinics, found that 36% had intermittent rhinitis, 64% persistent rhinitis, 59% mild rhinitis, and 41% moderate-severe rhinitis. Bachert et al. [16] assessed the data of 554 subjects recruited from the general population with rhinitis symptoms. They found that 59% had intermittent rhinitis, 41% persistent rhinitis, 25% mild rhinitis, and 75% moderate-persistent rhinitis. Bousquet et al. reported that 46% of 3,052 subjects had intermittent rhinitis, 54% persistent rhinitis, 19% mild rhinitis, and 81% moderate-severe disease [17]. Consistent with findings from previous surveys, it can be concluded that the prevalence of moderate to severe AR is higher among women than among men [1]. Likewise previous data, also this survey found high symptom burden among moderate to severe-, and a very good symptom control in mild AR patients who presented to their physician [13,16]. The most frequent patient-reported symptoms were: nasal congestion and obstruction as well as rhinorrhoea and ocular symptoms; in agreement with the results of Asia-Pacific Survey of Katelaris et al. [18].

Overall, there was only a poor correlation between the AR severity evaluated by physicians and that based on patients’ opinion. In good agreement with previous results of Canonica et al. [5] it can be concluded that physicians underrate the severity of AR in many cases. The underestimation of the severity may partially lead to adherence problems. Non-adherence to the prescribed therapeutic regimen is a worldwide problem. Reviews about various diseases conducted across countries are consistent in estimating non-compliance between 30 and 50% [19]. However, it is clear that adequate management of AR, including the patient’s adherence, is essential to achieve optimal therapeutic outcome. In our survey the compliance with tablet-taking and nasal spray use was found to be between 50 and 65%; mostly depending on the dosage form (higher for tablet-taking) and being fast independent from the severity of AR.

Several studies have confirmed the importance of rhinitis symptoms in the future development of asthma [20]. The severity of rhinitis may also affect the development of asthma. In the present study the prevalence of asthma was found to be 53.32% in the mild, while 57.52% in the moderate-severe AR group. Other authors have found disparate results according to the population studied. Marogna et al., in an epidemiologic study carried out in 832 subjects with intermittent rhinitis, found that 11.6% developed asthma when AR was mild and 22.2% when the condition was moderate-severe, whereas in 968 subjects who had persistent AR, 30.1% and 35.4% developed asthma when AR was mild and moderate-severe, respectively [3].

This is the only multicenter study ever conducted in Hungary to describe the symptoms, impact on everyday life and concomitant asthma frequency of AR; however as a real-life investigation it has many limitations. The bias on symptom severity caused by a possible not proper AR diagnosis or treatment non-adherence cannot be excluded in some cases. There were 74 surveys which were not included in the analysis due to missing data. Furthermore, some local and oral antihistamines, nasal decongestants may be purchased over-the-counter in pharmacies, therefore their use is completely unknown in this survey.

## Conclusion

In conclusion, this survey, enrolling 933 patients with AR presenting to their specialist and lead by the Hungarian Respiratory Society, found that a significant proportion of patients have moderate or severe disease, persistent symptoms and comorbidities such as asthma in Hungary. A more severe disease was shown to be more frequent among women. Furthermore, our results highlighted the poor symptom control of patients who presented with moderate or severe and persistent disease.

## Competing interests

The authors declare that they have no competing interests.
